# GRANDPARENTHOOD AND RISK OF MORTALITY: FINDINGS FROM THE HEALTH AND RETIREMENT STUDY

**DOI:** 10.1093/geroni/igz038.2340

**Published:** 2019-11-08

**Authors:** Lea Ellwardt, Karsten Hank, Carlos F Mendes de Leon

**Affiliations:** 1 University of Cologne, Cologne, Germany, North-Rhine Westphalia, Germany; 2 University of Cologne – Institute of Sociology & Social Psychology, North-Rhine Westphalia, Germany; 3 University of Michigan School of Public Health, Ann Arbor, Michigan, United States

## Abstract

Grandparenthood is a significant social role for older adults and may have important health implications. Parenthood itself has been associated with some protective health effects, although findings have been mixed. Whether grandparenthood is associated with important long-term health effects such as mortality is largely unknown. This study examines the grandparenthood-mortality nexus, and whether it is modified by gender and education. Longitudinal data from the Health and Retirement Study (HRS) were used, comprising twelve biennial follow-up waves from 1992 to 2014 with linked data on vital status derived from the National Death Index. Submodules assessed participants’ family structure during follow-up. The sample included 24,325 participants aged > 51 years with at least one child. Cox proportional hazard models were used to test the association between grandparenthood and mortality risk with adjustment for socio-demographic variables, for social variables including characteristics of and contact with children, and for health variables, including measures of overall, functional and mental health. Stratified models assessed these associations separately by gender and education. Grandparenthood was associated with a substantially increased mortality risk in women (fully adjusted HR = 1.65; 95% CI 1.27-21.14), and increased with larger number of grandchildren. No significant association was found for men (fully adjusted HR=1.25; 95% CI 0.97-1.62). Mortality risks associated with grandparenthood were highest among grandparents with low levels of education. The findings are among the first to suggest a potential grandparenthood survival “penalty”, especially for grandmothers. Higher levels of education appear to mitigate this negative survival effect among grandparents.

